# Intestinal Tissues Induce an SNP Mutation in *Pseudomonas aeruginosa* That Enhances Its Virulence: Possible Role in Anastomotic Leak

**DOI:** 10.1371/journal.pone.0044326

**Published:** 2012-08-31

**Authors:** Andrea D. Olivas, Benjamin D. Shogan, Vesta Valuckaite, Alexander Zaborin, Natalya Belogortseva, Mark Musch, Folker Meyer, William L.Trimble, Gary An, Jack Gilbert, Olga Zaborina, John C. Alverdy

**Affiliations:** 1 Department of Surgery, University of Chicago, Illinois, United States of America; 2 Department of Medicine, University of Chicago, Chicago, Illinois, United States of America; 3 Institute for Genomic and Systems Biology, Argonne National Laboratory, Argonne, Illinois, United States of America; 4 Bioengineering Institute for Advanced Surgery and Endoscopy, Chicago, Illinois, United States of America; The Scripps Research Institute and Sorrento Therapeutics, Inc., United States of America

## Abstract

The most feared complication following intestinal resection is anastomotic leakage. In high risk areas (esophagus/rectum) where neoadjuvant chemoradiation is used, the incidence of anastomotic leaks remains unacceptably high (∼10%) even when performed by specialist surgeons in high volume centers. The aims of this study were to test the hypothesis that anastomotic leakage develops when pathogens colonizing anastomotic sites become *in vivo* transformed to express a tissue destroying phenotype. We developed a novel model of anastomotic leak in which rats were exposed to pre-operative radiation as in cancer surgery, underwent distal colon resection and then were intestinally inoculated with *Pseudomonas aeruginosa,* a common colonizer of the radiated intestine. Results demonstrated that intestinal tissues exposed to preoperative radiation developed a significant incidence of anastomotic leak (>60%; p<0.01) when colonized by *P. aeruginosa* compared to radiated tissues alone (0%). Phenotype analysis comparing the original inoculating strain (MPAO1- termed P1) and the strain retrieved from leaking anastomotic tissues (termed P2) demonstrated that P2 was altered in pyocyanin production and displayed enhanced collagenase activity, high swarming motility, and a destructive phenotype against cultured intestinal epithelial cells (i.e. apoptosis, barrier function, cytolysis). Comparative genotype analysis between P1 and P2 revealed a single nucleotide polymorphism (SNP) mutation in the *mexT* gene that led to a stop codon resulting in a non-functional truncated protein. Replacement of the mutated *mexT* gene in P2 with *mexT* from the original parental strain P1 led to reversion of P2 to the P1 phenotype. No spontaneous transformation was detected during 20 passages in TSB media. Use of a novel virulence suppressing compound PEG/Pi prevented *P. aeruginosa* transformation to the tissue destructive phenotype and prevented anastomotic leak in rats. This work demonstrates that *in vivo* transformation of microbial pathogens to a tissue destroying phenotype may have important implications in the pathogenesis of anastomotic leak.

## Introduction

When patients undergo removal (resection) and re-connection (anastomosis) of a segment or whole portion of the gastrointestinal tract, a significant number will develop anastomotic leaks despite being operated on by highly qualified surgeons in high volume centers [1]. Anastomotic leaks cause major long term bowel dysfunction (incontinence), high cancer recurrence rates, decreased long term cancer survival and sepsis- related deaths [2], [3]. The cause of anastomotic leaks remains unknown.

Cohn first proposed in 1955 that the microbial content of the gut plays a central role in the pathogenesis of anastomotic leak [4]. In his experiments, dogs were subjected to colon anastomosis and division of the mesenteric blood vessels to cause ischemia and delayed healing. One group was administered intraluminal antibiotics (tetracycline directly into the bowel via an indwelling catheter) and the other saline. Antibiotic treated dogs demonstrated complete anastomotic healing and recovery whereas those administered saline developed major leakage with peritonitis and death. Shardley was the first to suggest that *P. aeruginosa* might play a causative role in anastomotic leak [5], and performed the first randomized prospective placebo blinded trial with antibiotics confirming a role for microbes in human anastomotic leak [6]. Yet despite this and other similar compelling observations, a microbial mechanism for anastomotic leak is generally not accepted, and around the world, anastomotic leak is posited to be primarily a problem of poor technique and/or poor wound healing [7]–[9].

Here we demonstrate that intestinal *P. aeruginosa,* now emerging as a frequent commensal in hospitalized patients following surgery, undergoes a stable genetic mutation at the site of tissue injury (i.e. anastomosis) that results in its transformation to a tissue destructive phenotype capable of causing anastomotic leak. Sequence analysis of *P. aeruginosa* recovered from the anastomosis site demonstrated a SNP in the *mexT* gene that confers swarming capacity, enhanced collagenase activity, and an epithelial disruption phenotype. The enhanced virulence phenotype was inducible by incubating the original strain with *ex vivo* anastomotic tissues demonstrating the importance of the *in vivo* environment and tissue injury for the expression of the tissue destroying phenotype. Use of polyethylene glycol polymers with added phosphate that we have previously shown suppress virulence in *P. aeruginosa* without affecting its growth, prevented its virulence transformation and prevented anastomotic leak. Therefore the aims of this study were to define the role of *P. aeruginosa* in anastomotic leakage in rats following colorectal surgery undergoing adjunctive pre-operative radiation, as occurs clinically, and to investigate the mechanism by which enhanced bacterial virulence disrupts healing anastomotic tissues.

## Materials and Methods

### Bacterial Strains

*Pseudomonas aeruginosa* strain MPAO1 obtained from the transposon mutant library at the University of Washington was used for initial inoculation in rats and is herein designated as the P1 strain. The transformed strain harvested from leaking rat anastomoses was designated a P2 as it is derived from the original MPAO1 strain (see results below). P1 and P2 strains were used in the comparative *in vitro* experiments. For each experiment, strains were directly cultured from a 10% glycerol stock stored at −80°C onto tryptic soy broth (TSB) agarized plates, and incubated at 37°C; overnight growing cells were used in all experiments accordingly to the respective design.

### Rat Model of Colorectal Anastomotic Leak

**Figure 1:**
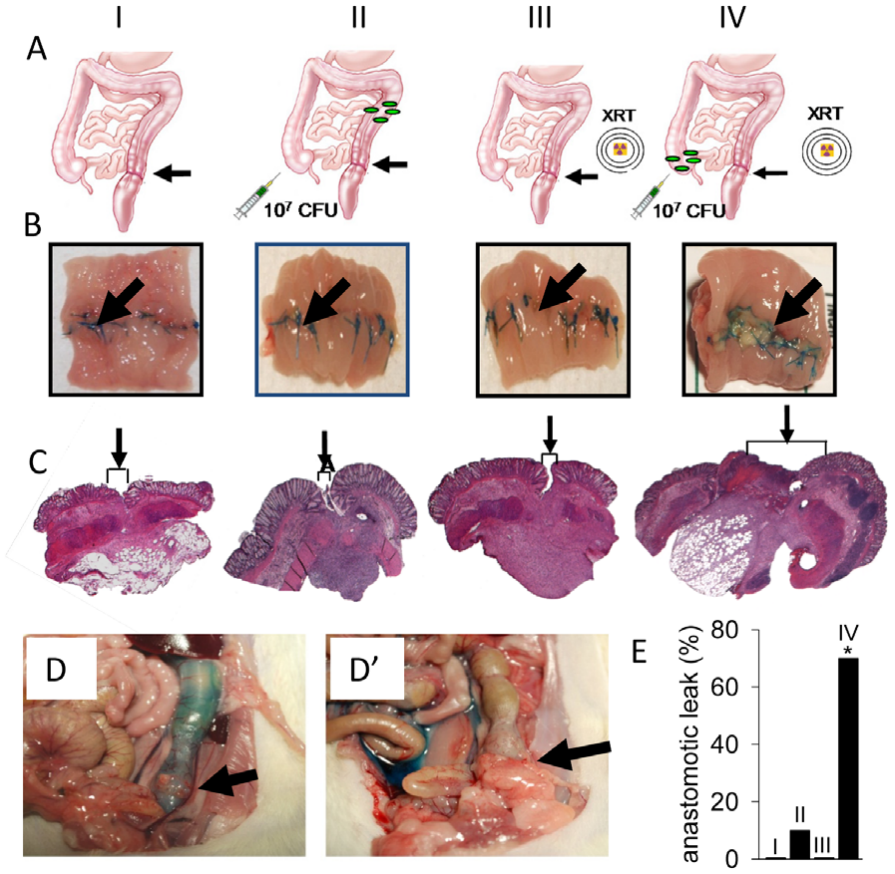
Anastomotic leak in rats exposed to pre-operative radiation and intestinal *P. aeruginosa*. (A) Sketch of anastomosis model and treatment groups. Treatment groups: I, anastomosis only; II, anastomosis + cecal injection of *P. aeruginosa* MPAO1, 10^7^ CFU; III, radiation + anastomosis; IV, radiation +anastomosis + cecal injection of *P. aeruginosa* MPAO1 (10^7^ CFU). Black arrows indicate the anastomotic site. (B) Excised and exposed suture lines of anastomotic sites. All suture lines are grossly intact except for group IV where ulceration/dehiscence is noted by the black arrow. (C) H&E staining of anastomotic tissues. Arrows and brackets indicate width of tissue apposition at suture line. (D, D’, E) Methylene blue assessment of anastomotic integrity demonstrating rare to no leaks in groups I–III (D) and gross extravasation in group IV (D’). Arrows indicate the site of anastomosis. (E) Incidence of anastomotic leak between groups. n = 12 (group I), n = 16 (group II), n = 9 (group III), n = 18 (group IV), *p<0.01.

All experiments were approved by the Institute for Animal Care and Use Committee at the University of Chicago. All studies involving mice conformed to the Animal Welfare Act and NIH Guidelines for the care and use of animals in biomedical research and with the University of Chicago Carlson Veterinary guidelines. Mice were housed in the animal facility at the University of Chicago. This facility has all the necessary personnel (veterinarians and support staff) and experience to handle the animals in accordance with Federal Regulations. All live infections in mice were performed in a class II biosafety cabinet in the biohazard facility. The method of euthanasia was consistent with the recommendations of the Panel on Euthanasia of the American Veterinary Medical Association and received approval by the University of Chicago IACUC. Every effort to avoid discomfort, distress, pain and injury was made in accordance with the conduct of scientifically sound research. Adult, male Wistar rats 300–350 g (Charles River Laboratory) were used for all experiments. Animals were allowed unrestricted access to rat chow and tap water throughout the experiments. In order to mimic the clinical practice of surgery for rectal cancer, rats were subjected to pre-operative fractionated pelvic radiation followed by a low colorectal resection and anastomosis. Prior to irradiation rats were sedated (40–80 mg/kg ketamine, 5–10 mg/kg xylazine; intraperitoneal injection, IP), and then placed in the supine position beneath the radiation cone. A total of 25 Gy of radiation, fractionated over 5 consecutive days (5 Gy per day; 1.47 Gy per minute), was delivered to the sigmoid colon and rectum using a Phillips RT250 x-ray generator. All other abdominal organs were excluded from the radiation field using a lead shield. One week following the last day of irradiation, rats were subjected to a laparotomy using aseptic technique and a 0.5 cm segment of colon at the peritoneal reflection was resected and an end-to-end rectosigmoid anastomosis was performed using 13 interrupted 6-0 Prolene sutures. After anastomosis formation, integrity was confirmed in all cases using a 5 ml saline rectal enema. In order to mimic nosocomial bowel contamination by *Pseudomonas aeruginosa,* an overnight culture of strain MPAO1 (200 µl of 10^7^ CFU in 10% glycerol) was directly injected into the cecum with a 25-gauge needle. The abdomen was closed in 2 layers using 4-0 Vicryl. Four groups of rats were studied: rats subjected to resection and anastomosis only (Group I), rats subjected to resection and anastomosis + cecal *P. aeruginosa* (Group II), rats subjected to preoperative radiation + resection and anastomosis (Group III), and rats subjected to preoperative radiation + resection and anastomosis + cecal *P. aeruginosa* (Group IV) (Fig. 1A). On postoperative day 6, all animals were euthanized and the anastomotic site evaluated for gross leakage using a 5 ml rectal methylene blue enema followed by excision of the cecum and anastomotic segment for microbial and histologic examination.

### Prevention of P. aeruginosa-mediated Anastomotic Leak with Topical Phosphate + PEG (PEG/Pi)

5% PEG 15–20 dissolved in 25 mM potassium phosphate buffer, pH 6.0, was administered via rectal enema (5 ml) to the anastomotic suture site at the end of the surgery.

### Histology

For histological evaluation, a 5 mm×5 mm segment of tissue centered at the anastomotic suture line was removed and fixed in formalin overnight at 4°C. Each tissue segment was then embedded in paraffin with the suture line mounted vertically within the block, cut into 5 µm sections, and stained with hematoxylin and eosin. Light microscopy was performed using a Zeiss Axioskop and images where captured using a Zeiss Axiocam digital color camera (1.25x magnification).

### Scanning Electron Microscopy (SEM)

To prepare for imaging with SEM, tissues were dissected into ice cold PBS, transferred to 4% paraformaldehyde Solution (USB 19943), and gradually dehydrated in 25% EtOH-PBS, 50% EtOH-PBS, 75% EtOH-PBS, 90% EtOH-PBS, and 100% EtOH for 40 minutes per each step. The samples were then transferred to 50% EtOH-HMDS (Hexamethyldisilazane Ted Pella 18605) for 1 hr and then 100% HMDS for an additional hour. Next, samples were transferred to freshly prepared 100% HMDS and maintained overnight in the hood to ensure evaporation. Samples were then fixed to a carbon stubs (Ted Pella 16111-9, Specimen mounts, Aluminium, 9 mm high, Ted Pella Carbon tape 9 mm, 16084-3), sputter coated with 80%Pt/20%Pd to 12 nm with Cressington Sputter Coater 208HR, and viewed in Fei Nova Nano SEM200.

### Wound Healing Assay

For wound healing experiments, IEC-18 cells (ATCC, Cat# CRL-1589) were seeded onto collagen-coated plastic p35 dishes and grown to confluent monolayers. Monolayers were scratched with a 10 µl pipette tip, incubated for 1 hour at 37°C, and the initial wound width was then measured. 200 µl of *P. aeruginosa* strains (MPAO1-P1 or MPAO1-P2, OD = 0.5) were added to culture dishes followed by incubation for 24 hours at 37°C, and the wound width was then re-measured. For experiments involving PEG/Pi treatment, cell medium was removed from wounded cells after 1 hour of incubation and replaced with 2 ml of 5% PEG dissolved in DMEM media supplemented with 25 mM phosphate buffer, pH 6.0) and incubated for 1 hour at 37°C, after which media was replaced with antibiotic-free DMEM media followed by bacterial inoculation as described. The values were expressed as the percentage of the initial wound healed.

### LDH Release

To quantitatively measure cell lysis, the amount of lactate dehydrogenase (LDH**)** released from the cells was measured after 24 hours using CytoTox 96 Cytotoxicity Assay (Promega, Madison, WI).

### *Caenorhabditis Elegans* Killing Assay

The *C. elegans* assay was performed as previously described [10], [11] with slight modifications to include a shorter pre-fasting procedure that made *C. elegans* less susceptible to infection. Briefly, synchronized L4-young adult nematodes were transferred from *E.coli* OP50 stock plates onto plain agarized plates, followed by a second transferring onto new plain agarized plates (60 mm diameter, Falcon). Next 1 ml of 100 µg/ml kanamycin was poured on the agar surface, and after 3 hrs worms were re-transferred to experimental *P. aeruginosa* lawns (MPAOP1 or MPAO1-P2) grown on NGM low phosphate agarized media (agar 17 g/L (Fisher), peptone 2.5 g/L (Sigma), cholesterol 5 mg/L (Sigma), NaCl 3 g/L, MgSO_4_ 1 mM, CaCl_2_ 1 mM, ampicillin, 40 µg/ml). Plates with *P. aeruginosa* were incubated overnight at 37°C, adjusted to room temperature for 1 hr, seeded with 5 pre-starved worms in 5 replicates per experiment performed, and incubated at 23°C. Mortality of worms was then followed dynamically for 60 hr.

### Random Amplified Polymorphic DNA Fingerprint Analysis

To verify that the *P. aeruginosa* strain recovered from the tissue of our experimental animals was of a similar genetic background as the stock lab strain MPAO1, random amplified polymorphic DNA (RAPD) PCR fingerprinting was used as previously described [12]. DNA was isolated from the stock PAO1 strain and *P. aeruginosa* strain recovered from the anastomoses of experimental animals using an Easy-DNA Kit (Invitrogen, Carlsbad, CA). Primers 208 (5′-ACGGCCGACC-3′) and 272 (5′-AGCGGGCCAA-3′), nucleotides producing reproducible polymorphisms with *P. aeruginosa*, were used for PCR.

### Collagenase Assay

Collagenase activity was assessed using an EnzChek Gelatinase/Collagenase Assay Kit (Molecular Probes, Eugene, OR). *P. aeruginosa* strains were grown overnight in liquid TSB media and then diluted 1∶100. For the assay, 180 µl of diluted bacteria in liquid TSB was added to 20 µl of collagen substrate (100 µg/ml; DQ collagen, type I from bovine skin, fluorescein conjugate; DQ collagen, type IV from human placenta, fluorescein conjugate). The negative control consisted of 180 µl TSB only added to 20 µl of collagen substrate. The reaction was measured every hour for 5 hours at an absorbance of 495 nm with a fluorescence microplate reader (FL x800, Bio-Tek Instruments Inc), where the increase in fluorescence measured is proportional to proteolytic activity. Values obtained for negative controls were subtracted from experimental samples to account for background fluorescence. All experiments were carried out in quintuplicate. At each time point, the OD of each sample was measured (at 600 nm) in order to normalize to the amount of bacteria in each sample.

### Apoptosis/Necrosis Assay

Rat intestinal epithelial IEC-18 cell were grown to a full confluence on Glass Bottom Culture Dishes (MatTek) in Dulbecco modified essential medium (DMEM) supplemented with 5% fetal bovine serum, 1% penicillin/streptomycin (Gibco), and 0.01 U/ml insulin. Then medium was replaced by antibiotic-free/FBS-free DMEM medium, and IEC-18 monolayers were infected with either P1 or P2 to reach final concentration of 1×10^6 ^cfu/ml. Cell were incubated for 3 hrs at 37C°, 5% CO_2_, followed by analysis for apoptosis and necrosis using Apoptic&Necrotic&Healthy Cells Quantification kit (Biotium, Inc.). Images were obtained with Axiovert 35 (Zeiss,Germany) fluorescent microscope. Semi-quantitative analysis was performed by counting apoptotic and necrotic cells, and the counts were normalized to the amount of nuclei stained by DAPI. 4 fields of ∼100 cells imaged from 4 independent dishes/group were included in the quantitative analysis.

### Tight Junction Assay

IEC-18 monolayers were prepared and infected as described above. After 3 hrs of co-incubation with P1 or P2, IEC-18 were washed twice with sterile PBS and fixed in 4% paraformaldehyde for 20 min at room temperature followed by PBS washes 3 times. Non-specific binding was blocked with blocking solution (1% BSA, 0,1% Triton X-100 in PBS) for 30 min. Tight junctions were labeled with rabbit anti-ZO-1 antibodies (1∶250) (Invitrogen) overnight at 4°C. Cells were washed three times with PBS and incubated for 1 h at RT with secondary anti-rabbit antibodies conjugated to Alexa Fluor 488 (1∶500) followed by PBS washes. Cells were visualized using a Leica DMIRE2 fluorescence microscope, SP2 laser scanning confocal (Leica microsystem, Mannheim, Germany).

### Swarming Motility

Medium for swarming motility assay consisted of 20 mM NH_4_Cl, 12 mM Na_2_PO_4_, 22 mM KH_2_PO_4_, 8.6 mM NaCl, 1 mM MgSO_4_, 1 mM CaCl_2_, 11 mM dextrose, 0.5% casamino acids, 0.5% Bacto-agar (Difco) as previously described [13]. Plates were allowed to dry overnight at room temperature and then bacterial strains were inoculated from overnight grown PIA plates onto the swarming plates using a sterile toothpick followed by incubation at 30°C.

### Pyocyanin Production

In liquid media, pyocyanin was extracted into chloroform followed by re-extraction into 0.2N HCl, in which it was quantitated by the absorbance at 520 nm normalized to bacterial cell density similar to that we described previously [10], [14]. In agarized media, equal square pieces of agarized media with lawns (diameter 2 cm) were extracted from plates, bacteria cells were removed in 1 ml of 0.9% NaCl, and pyocyanin was extracted from homogenized agar by chloroform followed by re-extraction into 0.2N HCl and measuring at 520 nm. Measurements were normalized to cell density measured by absorbance at 600 nm in 0.9% NaCl solutions.

### Transformation of MPAO1 to the MPAO1-P2 Phenotype in ex vivo Experiments

Laparotomy and low colorectal anastomosis formation were performed in animals that were either exposed or not exposed to preoperative pelvic radiation as described above. On postoperative day 1, all animals were sacrificed and the anastomotic segment and cecum (1 cm segment) were removed. Tissue segments (cecum, colon anastomosis) were homogenized in 1 ml sterile saline, incubated with strain MPAO1, and grown under static conditions, at 37°C. On day 3 of incubation, aliquots were plated onto Pseudomonas isolation agar (PIA) and 100 individual colonies were sub-cultured and evaluated for swarming motility. In selected experiments, tissue segments were homogenized in 1 ml of 5% PEG/Pi.

### Genome Sequencing

Genomic DNA sequencing was performed on both MPA01-P1 and MPA01-P2 using the Illumina GAIIx sequencer to a total of 137 and 115 fold coverage, respectively. Three libraries were created for each strain: 1×36 bp (370/340 million bp), 2×100 bp PE mate pairs with a 300 bp insert (320/180 Mbp) and 2×100 bp PE mate pairs with a 3000 bp insert (380/420 Mbp). All libraries were quality controlled and assembled de-novo using the mira assembler [15]. The MPA01-P1 and MPA01-P2 assemblies contained 6.26 Mbp and 6.29 Mbp, in 125 and 363 contigs respectively. These MPA01-P1 and MPA01-P2 assemblies had 99.99 and 99.95% nucleotide identity to the PAO1/DSM-1707 strain previously described [16]. The strain genomes were aligned to the reference strain NC_002516 [17] using BRESEQ 0.13 (Barrick, J. BRESEQ, unpublished software http://barricklab.org/breseq and to each other to determine areas of difference. The reads are deposited in SRA as accession SRA049017. These Whole Genome Shotgun projects have been deposited at DDBJ/EMBL/GenBank under the accessions AHKM00000000 and AHKN00000000. The version described in this report is the first version, AHKM01000000 and AHKN01000000.

### Replacement of mexT in the P2

The lambda Red-based technique modified for *P. aeruginosa* [18] was used for replacement of *mexT* in P2 strain. The entire coding region of *mexT* from MPA01 was amplified using primers forward 5′ CGG ATA ATG ATC GGG GGT AT 3′ and reversed 5′ CCG AAT TTT TCC AGC TCC TC 3′, and 10 µl of amplified *mexT*-P1 was directly transformed in MAPO1-P2/pUCP18-RedS electrocompetent cells. Transformants were selected by plating on PIA containing 300 µg/ml of chloramphenicol. A cure of the plasmid was achieved on plates containing 10% sucrose. Transformants were verified for correct PCR insertion by *mexT* sequencing using primers Forward 5′ GCC TGT CAG TGA TCC TAT GC 3′ and Reversed 5′ GAT CGC CGA TGA ACA TGC 3′.

### Statistical Analysis

Statistical analysis of anastomotic leak rate was performed using Fisher’s Exact Test (Prism software). Significance for *ex vivo* studies was determined using 1-way ANOVA analysis. All other non-parametric data were analyzed using Kruskal Wallis and Mann-Whitney tests. Kaplan-Maier survival graph was analyzed using SPSS software. Significance was determined as a p-value <0.05.

## Results

### Intestinal Exposure to Radiation and *P. aeruginosa* Causes Spontaneous Anastomotic Leak in Rats

**Figure 2:**
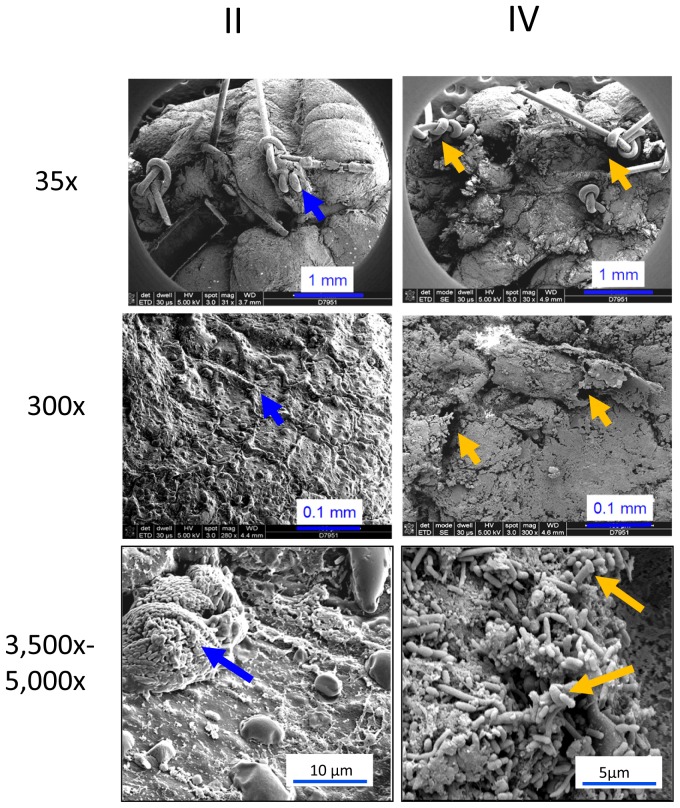
Scanning electron microscopy (SEM) images of anastomosis tissues. Blue arrows indicate healed anastomosis (30x), intact intestinal epithelium (300x), and macrophages on epithelial surface (3,500x) in group II. Orange arrows indicate discontinuity near or at the anastomosis (30x), disrupted intestinal epithelium (300x), and a high degree of bacterial colonization/adherence at the edge of non-healed anastomoses (5,000x). 50 images from each group of 5 mice were obtained, and representative images are displayed.

In order to define the role of *P. aeruginosa* on anastomosis healing, we first developed a novel anastomosis model in which rats were subjected to preoperative fractioned radiation similar to clinical practice. Rats then underwent distal colon resection and anastomosis followed by intestinal inoculation with *P. aeruginosa* via cecal puncture at the end of the operation (Group IV) (Fig. 1A). Control groups included: rats subjected to resection and anastomosis only (Group I), rats subjected to resection and anastomosis + cecal *P. aeruginosa* (Group II), rats subjected to preoperative radiation + resection and anastomosis (Group III). When we tested and directly examined the anastomoses of all rats on postoperative day (POD) six, rats in group IV demonstrated evidence of a significant incidence of spontaneous anastomotic leak with grossly visible disruption of the anastomotic suture line (Fig. 1B), dense adhesions to the anastomosis, immune cell infiltration (Fig. 1C, Fig. S1), and gross extravasation of injected intraluminal (rectal) contrast material (methylene blue) (Fig. 1D’). A major distinguishing characteristic in well healing and intact anastomoses appeared to be the absence of dense adhesions to the external aspects of the anastomotic suture line, grossly intact and visible anastomotic healing both externally and internally, and lack of extravasation when intraluminal contrast was injected. Gross histology confirmed these findings. When we opened the anastomoses and visualized the mucosa, anastomotic suture lines were intact in healed anastomosis but demonstrated focal disruptions in those that leaked. Scanning electron microscopy (SEM) of the mucosal of anastomotic suture lines in rats exposed to *P. aeruginosa* (groups II and IV) (Fig. 2) demonstrated in group II: an intact suture line at the anastomosis (35x), a smooth intact appearance of the epithelial surface (300x), and the presence of an immune cells (macrophages) and few if any bacteria (3500x); in group IV: the suture line appears disrupted (35x), there is disintegration of the epithelial surface (300x), and there are abundant microbial cells at sites of disruption (3500x). Taken together these findings demonstrate that when *P. aeruginosa* is introduced to the proximal colon of rats subjected to preoperative radiation and a distal colon anastomosis, it appears to adhere to the anastomotic sites and cause leakage.

### *P. aeruginosa* Isolated from a Disrupted Anastomosis Displays a Tissue Destroying Phenotype

**Figure 3:**
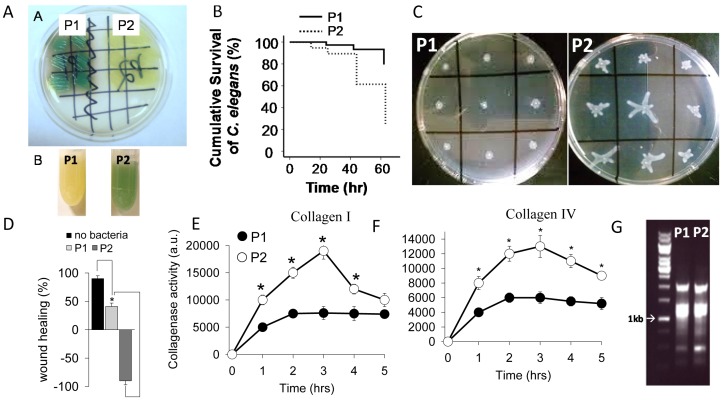
P1 and P2 phenotypes of *P. aeruginosa* MPAO1. (A) Pyocyanin production seen as green color pigmentation on solid PIA and liquid TSB media. (B) Kaplan-Meyer survival curves of *C. elegans* N2 feeding on P1 and P2. Cumulative survival is represented of 2 experiments, n = 7/dish, 5 dishes/experiment, p<0.01. (C) Swarming motility. (D) Wound healing assay. Wound width was calibrated and measured using the MicroSuite software for imaging applications (Olympus SZX16). Wound healing of −100% indicates a 2 fold increase in the wound width compared to the baseline width. n = 12, *p<0.01. (E,F) Collagenase activity of P1 and P2 measured by degradation of fluorescent labeled collagen I (E) and collagen IV (F) as substrates. n = 6, *p<0.01. Fluorescence values were normalized to cell density measured by absorbance at 600 nm. Results are representative of 3 independent experiments. (G) RAPD fingerprint analysis demonstrating a similar genetic background of the P1 and P2 phenotype strains.

We next tested the hypothesis that strains of *P. aeruginosa* isolated from a leaking anastomosis would display an enhanced virulence phenotype. Therefore we assessed the phenotype of *P. aeruginosa* recovered from leaking anastomotic sites in rats subjected to radiation exposure and intestinal inoculation with *P. aeruginosa* (group IV) and compared it to the inoculating MPAO1 strain. On postoperative day 6 (POD 6) leaking disrupted anastomoses were resected, homogenized and *P. aeruginosa* was recovered by plating tissues on Pseudomonas isolation agar (PIA). Hundreds of single colonies were directly chosen from the plates and re-plated (streaked) on fresh PIA plates. We found that majority of colonies displayed low pyocyanin production which we initially recognized as displaying attenuated virulence (Fig. 3A, top panel). To confirm this, we tested the virulence of these colonies in *C. elegans* and surprisingly observed enhanced killing of the yellow colonies compared to the initial strain (Fig. 3B). We also observed dendritic-like edges of lawns generated by the yellow colonies suggesting that they possess swarming motility. We therefore performed swarming assays and observed high swarming motility in these cells vs almost no swarming activity in the initial strain (Fig. 3C). The strains were then tested for their ability to disrupt cellular elements of anastomotic tissues (epithelial cells, collagen) and observed enhanced epithelial cell destruction (Fig. 3D) and a high level of collagenase (Fig. 3E,F) among the yellow appearing colonies. To verify that the strain recovered from leaking anastomotic tissues was genetically similar to the wt strain used for intestinal inoculation (MPAO1) and not a pre-existing commensal in the rat gut, we performed genetic fingerprint analysis (RAPD) that confirmed their similarity (Fig. 3G).

**Figure 4:**
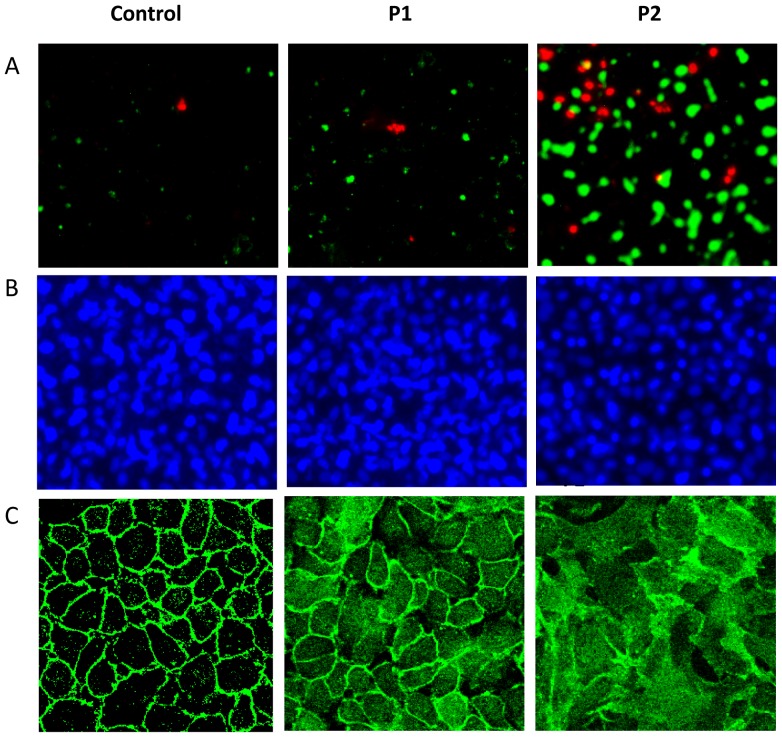
P2 induces significant apoptosis and structural changes in the tight junction protein ZO-1 in in IEC-18 monolayers. (A) IEC-18 cells infected with P1 and P2 for 3 hrs were analyzed for apoptosis and necrosis with Apoptic&Necrotic&Healthy Cells Quantification kit (Biotium, Inc.) using fluorescence microscope Axiovert 35 (Zeiss,Germany). FITC-Annexin V (apoptotic cells, green), EtD-III (necrotic cells, red). (B) Staining of nuclei with Hoechst 33342. (C) IEC-18 monolayers treated with antibody to ZO-1.

Surprisingly, we observed that virtually all colonies isolated from disrupted anastomoses were attenuated in the production of the toxic metabolite pyocyanin grown on PIA at high cell density. Conversely, when grown in liquid culture, there was an increase in pyocyanin production compared to MPAO1 (Fig. 3A bottom panel, and Fig. S2). Spontaneous conversion of P1 and P2 *in vitro* was tested by subculture to 20 passages of each in rich nutrient TSB media. No P2 phenotype was detected in subcultures of P1 and conversely no P1 was detected in subcultures of P2, and the pattern of pyocyanin production by P2 (low on PIA, high in liquid TSB) was stably reproduced. We named the isolate from disrupted anastomotic tissues MPAO1-P2 (herein termed P2) and the initial inoculating strain MPAO1-P1 (herein termed P1). The ability of P2 to degrade collagens demonstrates its ability to cause a full thickness defect at the site of anastomotic injury. Next we assessed the effect of P2 on apoptosis/necrosis and tight junctional integrity of cultured epithelial monolayers. Rat intestinal epithelial EIC-18 cells were infected with 10^6^ CFU P1 or P2 and incubated for 3 hrs followed by staining for apoptosis (FITC-Annexin V, green fluorescence), necrosis (EtD-III, red fluorescence), nuclei (Hoechst 33342, blue), and tight junction (ZO-1 immunostaining). Using confocal microscopy, we observed significant apoptotic cells in IEC-18 cells infected with P2 compared to P1 (Fig. 4A, and Fig. S3 demonstrating quantitative analysis of apoptotic cells), while nuclei staining demonstrated the same amount of the cells in both groups (Fig. 4B). About 10% of IEC-18 cells appear to be necrotic at 3 hrs when exposed to P2 infection confirming its cytotoxicity (Fig. 4A). P2 also caused a striking loss of tight junction integrity as judged by ZO1 staining (Fig. 4C). Taken together these results demonstrate that the P2 phenotype expresses a degree of virulence that is sufficient to disrupt healing anastomotic tissues from the most superficial elements (epithelia) to the submucosa and serosa (collagen).

### Regional Distribution of P1/P2 within the Rat Colon

To determine the relative distribution of P1 versus P2 in the two treatment groups in which *P. aeruginosa* was injected into the cecum (groups II, IV), we selectively cultured for *P. aeruginosa* from the cecum and anastomotic sites and then assessed retrieved strains for swarming. Results demonstrate that cecal *P. aeruginosa* from both groups displayed a low incidence of the P2 phenotype (<10% of total recovered colonies). In contrast, a high incidence (>80%) of the P2 phenotype was recovered (by culture) at anastomotic sites in both non-radiated (group II) and radiated (group IV) rats suggesting that surgical injury plays a role in the transformation to, or selection for P2.

### Transformation of P1 to P2 using ex vivo Intestinal Tissues

**Figure 5:**
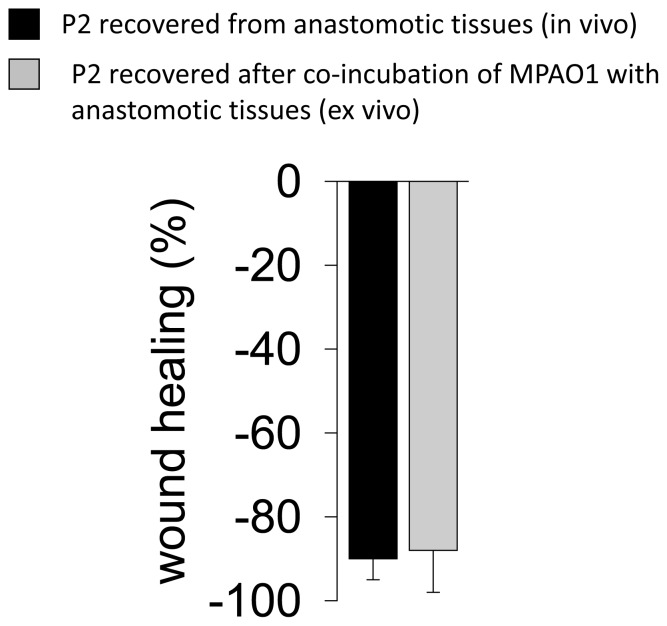
Wound healing assay. The P2 strain recovered from anastomotic tissues (*in vivo*) and the P2 strain recovered after co-incubation of MPAO1 with anastomotic tissues (*ex vivo*) similarly destroy wounded epithelial IEC-18 monolayers.

To determine if anastomotic tissues themselves can shift P1 to P2, we performed experiments in which P1 was exposed to rat colon tissues *ex vivo*. Tissues were obtained from reiterative studies in rats subjected to a colon resection and anastomosis with no exposure to *P. aeruginosa* and rats without anastomosis. Cecum and colon segments were excised and homogenized in sterile saline. The P1 strain was added to the homogenate and the solution incubated at 37°C. After 72 hours, *P. aeruginosa* was recovered on PIA, and then examined for pyocyanin production and swarming motility. 80–90% of recovered colonies from anastomotic tissues were found to produce the P2 phenotype. No transformation was detected when P1 was incubated in saline alone and ∼10% of colonies displayed the P2 phenotype when P1 was exposed to non-traumatized cecal tissues or non-anastomotic colon tissues. Comparative analyses of P2 transformed *in vivo* and P2 transformed *ex vivo* demonstrated similar ability to destroy wounded epithelial cell monolayers (Fig. 5). These findings suggested that factors present within colonic tissues themselves may be responsible for the shift of P1 to P2.

### SNP Mutation in mexT is Responsible for P2 Phenotype

**Figure 6:**
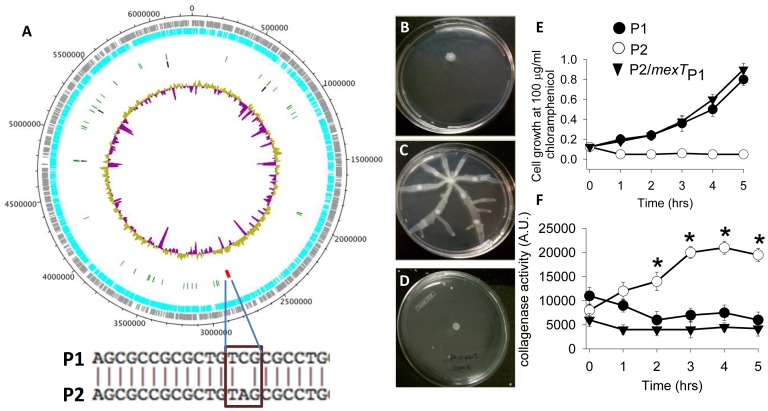
SNP mutation in *MexT* is responsible for P2 phenotype. (A) Genome DNA sequence comparative map of *P. aeruginosa* MPAO1-P1 and MPAO1-P2 at the DSM-1707 backbone annotated with the MexT locus. Grey and teal bands: annotated coding regions; red tick: location of MexT locus; green ticks: tRNAs; black ticks: rRNAs; inner circle GC content. (B–D) swarming motility in (B) MPAO1-P1 (P1), (C) MPAO1-P2 (P2), and (D) MPAO1-P2 in which *mexT* was replaced by *mexT* gene amplified from MPAO1-P1 (P2/*mexT*_P1_). (E) Growth curves at 100 µg/ml chloramphenicol demonstrating acquisition of chloramphenicol resistance in P2/*mexT*_P1._ (F) Collagenase activity measured by fluorescence of fluorescent labeled gelatin as a substrate. n = 6, *p<0.01. Results are representative of 3 independent experiments.

To determine if the observed shift from P1 to P2 was secondary to a genotypic change, genome sequencing was performed. A single nucleotide mutation (C→A position 2807731 in the NC002516 genome) localized in the *mexT* gene was identified (Fig. 6A). These results were further confirmed by direct sequencing of the amplified *mexT* in P1 and P2 strains using primers Forward 5′ GCC TGT CAG TGA TCC TAT GC 3′ and Reversed 5′ GAT CGC CGA TGA ACA TGC 3′. Sequencing of amplified *mexT* from P2 strain isolated *ex vivo* demonstrated the same C→A SNP.

The *mexT gene* is predicted to encode a full-length, 304-residue MexT protein, a regulator of the MexE-MexF-OprN multidrug efflux system of *P. aeruginosa* [19]. The genome sequence demonstrated the intact *mexT* in P1 (Protein accession number Pubseed:fig|6666666.7915.peg.414) [20] while the SNP (C→A) mutation in P2 was found to create an in-frame stop codon, limiting the possible products to a 44-residue presumed non-functional truncated protein (Pubseed: fig|6666666.7916.peg.276), and a 242-residue product resulting from re-initiation of translation at M63 (Protein accession number Pubseed:fig|6666666.7916.peg.277) (http://pubseed.theseed.org/seedviewer.cgi). (GenBank Protein accession number for the intact MexT protein from PAO1 is AJ007825.1).The functionality of MexT can be assessed by the level of resistance to chloramphenicol and fluoroquinolones antibiotics that depends on MexT-regulated expression of genes encoding the multi-drug efflux pump MexEF-OprN [21], [22]. We assessed antibiotic resistance of P1 and P2 and observed that the P2 strain showed a 10-fold higher sensitivity to norfloxacin compared to the P1 strain (MIC 0.38 µg/ml for P1 and 3.8 for P2) and ∼6 fold higher sensitivity to chloramphenicol (Fig. S4). To verify the SNP mutation on the phenotype shift, we replaced *mexT*-P2 by *mexT*-P1 in strain P2 to create P2/*mexT*_P1_. This replacement led to the reversion of P2 to P1 phenotype as determined by the pyocyanin production pattern (high pyocyanin on agarized TSB, low pyocyanin in liquid TSB), absence of swarming (Fig. 6B–D), high resistance to chloramphenicol (Fig. 6E), and attenuated collagenase activity (Fig. 6F). Taken together, these data suggest that the SNP mutation in *mexT* is responsible for transformation of the P1 to the P2 strain.

### Prevention of the P1→P2 Transformation is Associated with Complete Anastomotic Healing

**Figure 7:**
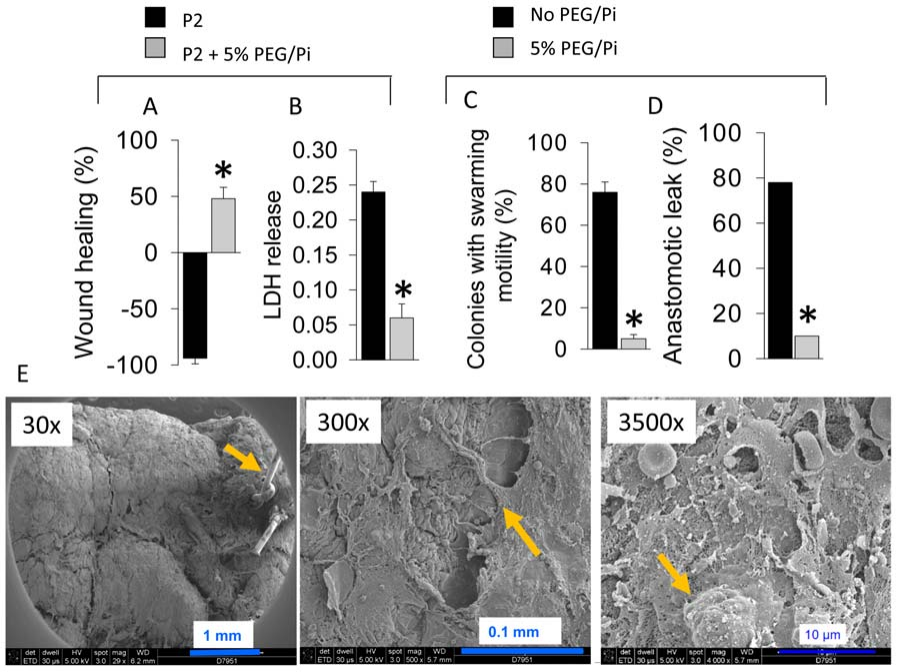
Protective effect of PEG/Pi. (A) Inhibition of wound apposition (healing) by *P. aeruginosa* MPAO1-P2. n = 5, *p<0.01. (B) Prevention of the cytotoxic effect of MPAO1-P2. n = 6, *p<0.01. (C) Frequency of transformation MPAO1 to P2 phenotype. n = 3, *p<0.01. (D) Frequency of anastomotic leak in rats. n = 10, *p<0.01. (E) SEM images of anastomotic tissues treated with 5% PEG/Pi demonstrating intact intestinal epithelium covered with a mucus-like film structure.

We previously demonstrated that a high molecular weight polyethylene glycol compound, PEG 15–20, attenuates virulence activation in *P. aeruginosa* in response to radiated epithelial cells and protects against post-radiated lethal sepsis [23]. In other work we demonstrated the importance of phosphate to prevent virulence activation and lethality in *P. aeruginosa* via its effect on phosphosensory and phosphoregulatory pathways that connect to quorum sensing. [10]-an effect that is maximized at a pH of 6.0 [11]. Therefore we prepared a 5% PEG15–20 solution in 25 mM potassium phosphate buffer at pH 6.0 (herein named PEG/Pi) and tested it in reiterative experiments. IEC-18 cells were pre-treated for 1 hr with 5% PEG/Pi prior to P2 inoculation. A marked protective effect against P2- induced cytotoxicity/disruption was observed. Epithelial monolayers remained intact as judged by LDH released and demonstrated cellular migration across the wound to 50% (Fig. 7A,B). 5% PEG/Pi also prevented the transformation of P1 to P2 during exposure to anastomotic tissues *ex vivo* (Fig. 7C). Finally, reiterative studies in rats exposed to pre-operative radiation followed by colon resection, anastomosis, and cecal injection with *P. aeruginosa* P1 (group IV rats) with 5% PEG/Pi given as an enema demonstrated significantly attenuated anastomotic leak rates compared to rats given rectal saline (Fig. 7D). The causality between the transformed P2 phenotype *in vivo* and anastomotic leak in this model is suggested by the observation no P2 strains were recovered from anastomotic tissues of rats treated with the PEG/Pi despite the compound having no microbicidal activity. This was confirmed by the SEM analysis of anastomotic tissues treated with 5% PEG/Pi that demonstrated absence of bacteria on the epithelial surface and healed epithelial surfaces (Fig. 7E). Taken together these results suggest that *in vivo* transformation of P1 to P2 is likely to play a key role in anastomotic leak in this model and may be prevented by virulence directed agents such as PEG/Pi.

## Discussion

Despite decades of refinements in technique, the development of ergonomic stapling devices, and the emergence of high volume super-specialized surgeons working in high volume centers, anastomotic leaks remain a real and present danger to patients. This is particularly evident in high risk areas of the gastrointestinal tract such as the esophagus and rectum where the incidence of leak persists at 10% and paradoxically appears to be increasing in incidence [24]–[27]. Results from the present study extend the observations by Cohn nearly 60 years ago [4] and later confirmed by Schardey in 1994 [5] and introduce a model which conforms to the molecular Koch’s postulates that microbial phenotype, rather than microbial presence alone, plays a role in the tissue disruption that characterizes anastomotic leak [1], [6], [28]. Placing this finding in the context of the practice of high risk gastrointestinal surgery is important as surgeons are operating on more complex and older patients who often received adjunctive chemotherapy and radiation either before or after intestinal resection. The promiscuous use of antibiotics may also contribute to colonization by more pathogenic strains of bacteria at anastomotic sites, that when properly cued by host elements at the site of tissue injury, can become transformed to express a tissue destructive phenotype. Such dynamic microbial virulence regulation that may be dependent on both spatial and regional context may explain, in part, the relative difficulty in predicting those at risk for anastomotic leak.

The ability of P1 to transform to P2 and acquire high swarming and enhanced ability to degrade collagen are undoubtedly important assets for *P. aeruginosa* to acquire as a mechanism to cause full thickness loss of anastomotic integrity. The data generated by the present study however are insufficient to determine the causative link between the P2 phenotype and anastomotic leak per se. Although anastomotic tissues, with or without exposure to radiation, induced the P2 phenotype, P2 may only exerts its full potential to cause leakage when it is further cued by radiated tissues- a hypothesis that currently cannot be tested *in vitro*. Evidence for this is our recent observation that *P. aeruginosa* virulence is activated by soluble factors released from radiated intestinal epithelial cells [23]. However there are major technical challenges to causally link the P2 phenotype to anastomotic leak. We introduced P2 into the cecum of rats with anastomotic construction but without radiation and it did not cause anastomotic leak (data not shown). In contrast to radiated rats, non-radiated rats maintain a normal microbiota and intact mucus layer which may shield against the virulence effects of the P2 phenotype. Additionally, once P2 is afforded the opportunity to adhere to anastomotic tissues in radiated mice, yet-to-be identified contact dependent host tissue factors may induce transcriptional changes in P2 that then confer an even more invasive tissue destroying phenotype. Laser capture transcriptome analysis of *P. aeruginosa* at the site of the anastomosis will be required to elucidate this potential mechanism.

Our justification for using MPAO1 is based on our previous work tracking dynamic virulence expression in *P. aeruginosa* in response to environmental stimuli and host tissue factors [10], [14], [29], [30]. At baseline MPAO1 is a low virulence expressing strain that in general requires exposure to local environmental cues and host tissue factors to cause severe injury such as lethality in worms (*C. elegans*) and gut- derived sepsis mice. As *P. aeruginosa* is one of the most common pathogens to colonize the gut following radiation, we felt it was best to model anastomotic leak with a strain that is well characterized, of low virulence potential at baseline, and with a publically available transposon library.

The discovery of the involvement of mexT in the P1 to P2 transition is intriguing. MexT has been described as a mutational “hot spot” in *P. aeruginosa* [16], [31]. MexT belongs to a LysR-type transcriptional regulator [19] whose expression determines the global transcription profile including MexEF-OprN efflux pump, quorum sensing system, and type III secretion system [21], [22], [32]–[34]. Strains of *P. aeruginosa* with functional MexT display *nfxC*-type antibiotic resistance that is characterized by increased resistance to chloramphenicol and fluoroquinolones [31], and the same profile we observed in the MPAO1 strain (P1). Among other phenotypic characteristics of *nfxC*-type resistant *P. aeruginosa* strains is abolished swarming motility [22] and attenuated pyocyanin, elastase, and rhamnolipids production [34]. Strain MPAO1 (P1 phenotype) displayed characteristics similar to that described for *nfxC*-type strains, however its pyocyanin production, although indeed delayed in liquid media, was enhanced on agarized media. Overall, *nfxC*-type strains are considered to display attenuated virulence. The known conversion between *nfxC* and non-*nfxC* strains is associated with the insertion of 8 bp sequence (CGGCCAGC) in *mexT* gene [19], [35]. The sequence of MPAO1 genome in the current study revealed that this strain (initially strain from Prof. Iglewski laboratory, later used as the parenteral strain for the transposon library) harbors the insertion of the 8 bp sequence in *mexT* gene that determines its P1 phenotype similar to that described by Kohler as the nfxc –type mutation [34]. The conversion of the P1 to the P2 however was not accompanied by the deletion of 8 bp but by the mutation C→A that reverted MexT back to the non-functional state, suggesting that several mechanisms of transformation exist. That the exact same mutation C→A that reverted MexT back to the non-functional state emerged in both *in vivo* (at the site of anastomosis) and *ex vivo* (co-incubation with anastomotic tissues) may indicate the presence of inducing factor (s) originating from the host, microbe, or their interaction. Paradoxically, non-*nfxC* strain harboring 8 bp insertion is considered as wt and *nfxC*-type strain with functional MexT is considered as a mutant [34]. Given that it would seem incongruous that a gene would emerge whose functionality is initially blocked, we considered that strains with the 8 bp insertion to be the mutant while the strain with the absence of this insertion as the wt strain. We assume that wtP1 switches to P2 via SNP mutation which is strongly selected for at the site of the anastomosis as an adaptive response to local microenvironmental conditions present as a result of tissue injury and radiation.

We ruled out the possibility of spontaneous conversion of P1 to P2 in the present study by subculturing both P1 and P2 strains for 20 passages in rich nutrient TSB media. No P2 phenotype was detected in P1 populations and no P2 spontaneously reverted to P1 in among 100 colonies selected for analysis at each point. We therefore concluded that the P1 to P2 conversion in the cecum (5–10%) and its high rate of conversion at anastomotic sites (>90%) is both a function of the *in vivo* environment per se as well as the tissue injury which obviously plays a more prominent role. It is important to keep in mind that the cecum of operated rats is still a stressed environment as the host has undergone general anesthesia and major intestinal surgery and metabolically is still in recovery phase. Therefore local cues within the cecum, albeit, less when compared to the anastomotic site, may induce or select for the P2 phenotype in this model as a result of the effects of systemic host stress.

In summary data from the present study support an evolving principle in clinical medicine that the gut represents a unique niche in which there is a spatialized ecologic feedback that leads to emergent traits among its colonizing microbes. In the ever changing chaos of this complex ecosystem, it is easy to imagine that a patient being prepared for intestinal cancer surgery with purgatives, antibiotics, radiation, and chemotherapy, who then undergoes a traumatic tissue injury while exposed to healthcare associated pathogens will harbor microbes whose virulence might be triggered by unique host and physico-chemical cues. The discovery of the P2 SNP confirms the importance of host factors as agents that play a key role in this response. Therapies that seek to target microbial virulence expression may have an ecological advantage over antibiotics that indiscriminately eliminate all potential pathogens and the protective microbiota with the real risk of the emergence of resistance. Further work will be necessary to unravel the molecular details of these events and to identify the microbial targets to develop novel anti- virulence compounds.

## Supporting Information

Figure S1 **Histological analysis of anastomotic tissues from rats of experimental groups II and IV.** M = mucosa; SM = submucosa; MP = muscularis propria; * = anastomosis.
(PDF)

Figure S2 **Pyocyanin production in agarized and liquid media.** TSB, tryptic soy broth. n = 5/group, *p<0.01.
(TIF)

Figure S3 **The percentage of apoptotic and necrotic IEC-18 cells co-incubated with** ***P. aeruginosa*** **of the P1 and P2 phenotypes.** The counts were normalized to the amount of nuclei stained by DAPI. 4 fields of ∼100 cells imaged from 4 independent dishes/group were included in the quantitative analysis.
(TIF)

Figure S4 **Chloramphenicol resistance of P1 and P2 strains.** Strains were cultured in TSB containing 250 µg/ml chloramphenicol and grown in 96-well plate (150 µl/well, shaking at 150 rpm, 37C°. Cell density was measured on Plate Reader at OD 600 nm. Values represent the mean of triplicate cultures.
(TIF)
