# Supplementary material for: Intestinal Tissues Induce an SNP Mutation in Pseudomonas aeruginosa That Enhances Its Virulence: Possible Role in Anastomotic Leak
Source: PLoS One. 2012 Aug 31;7(8):e44326. doi: 10.1371/journal.pone.0044326 (PMC3432121; doi:10.1371/journal.pone.0044326)
Supplement: Figure S1 — Histological analysis of anastomotic tissues from rats of experimental groups II and IV. M = mucosa; SM = submucosa; MP = muscularis propria; * = anastomosis. (PDF) [file pone.0044326.s001.pdf]

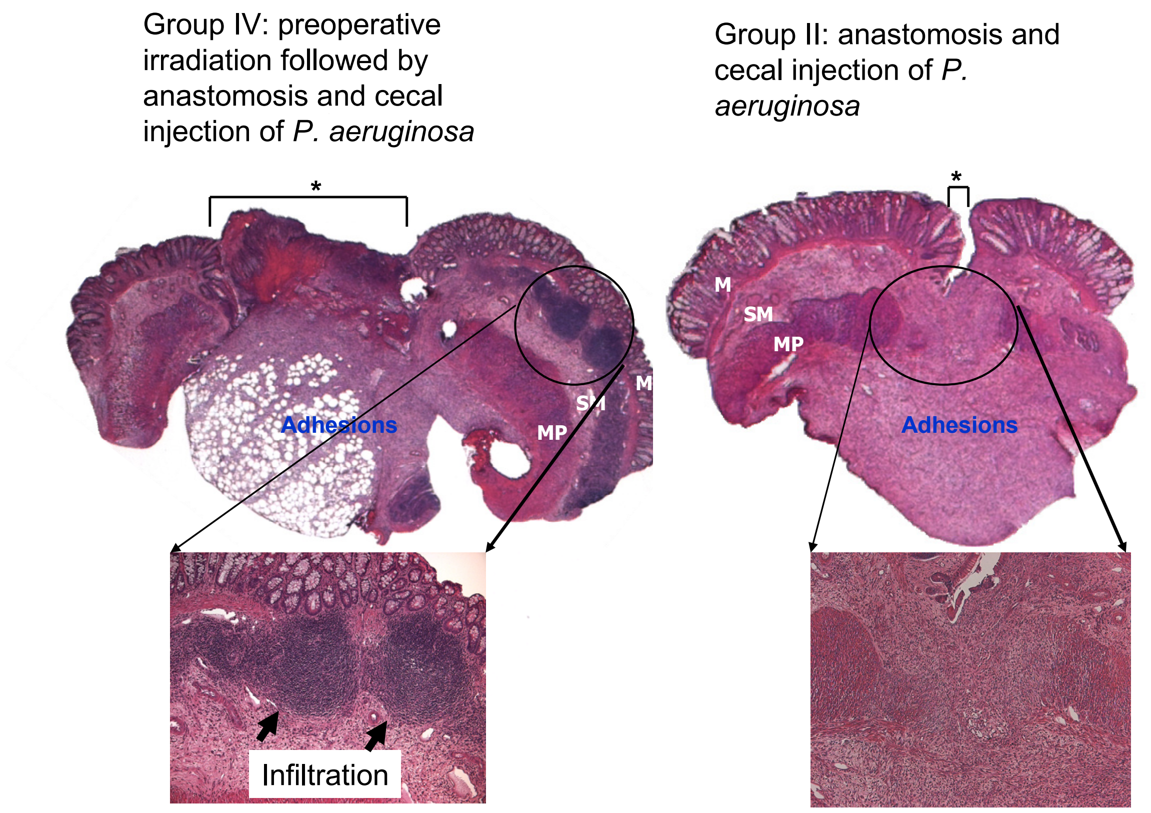

Supplemental Fig.S1. Histological analysis of anastomotic tissues from rats of experimental groups II and IV. M= mucosa; SM= submucosa; MP= muscularis propria; * = anastomosis.
