# Supplementary figures and images for: Intestinal Tissues Induce an SNP Mutation in Pseudomonas aeruginosa That Enhances Its Virulence: Possible Role in Anastomotic Leak
Source: PLoS One. 2012 Aug 31;7(8):e44326. doi: 10.1371/journal.pone.0044326 (PMC3432121; doi:10.1371/journal.pone.0044326)

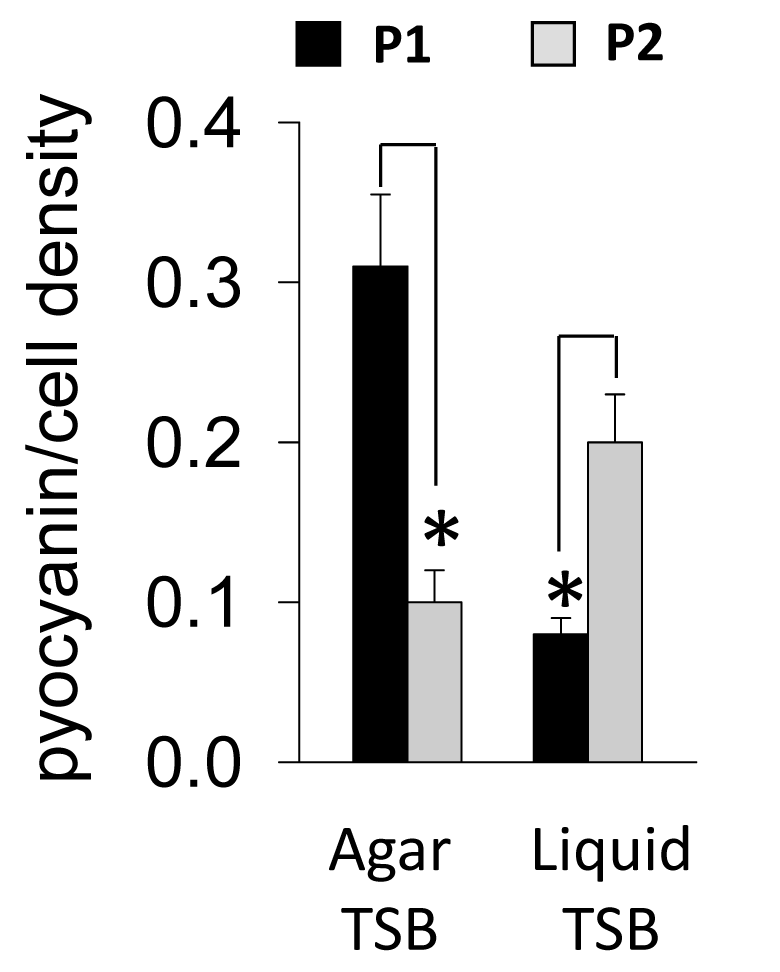

Supplement: Figure S2 — Pyocyanin production in agarized and liquid media. TSB, tryptic soy broth. n = 5/group, *p<0.01. (TIF) [file pone.0044326.s002.tif]

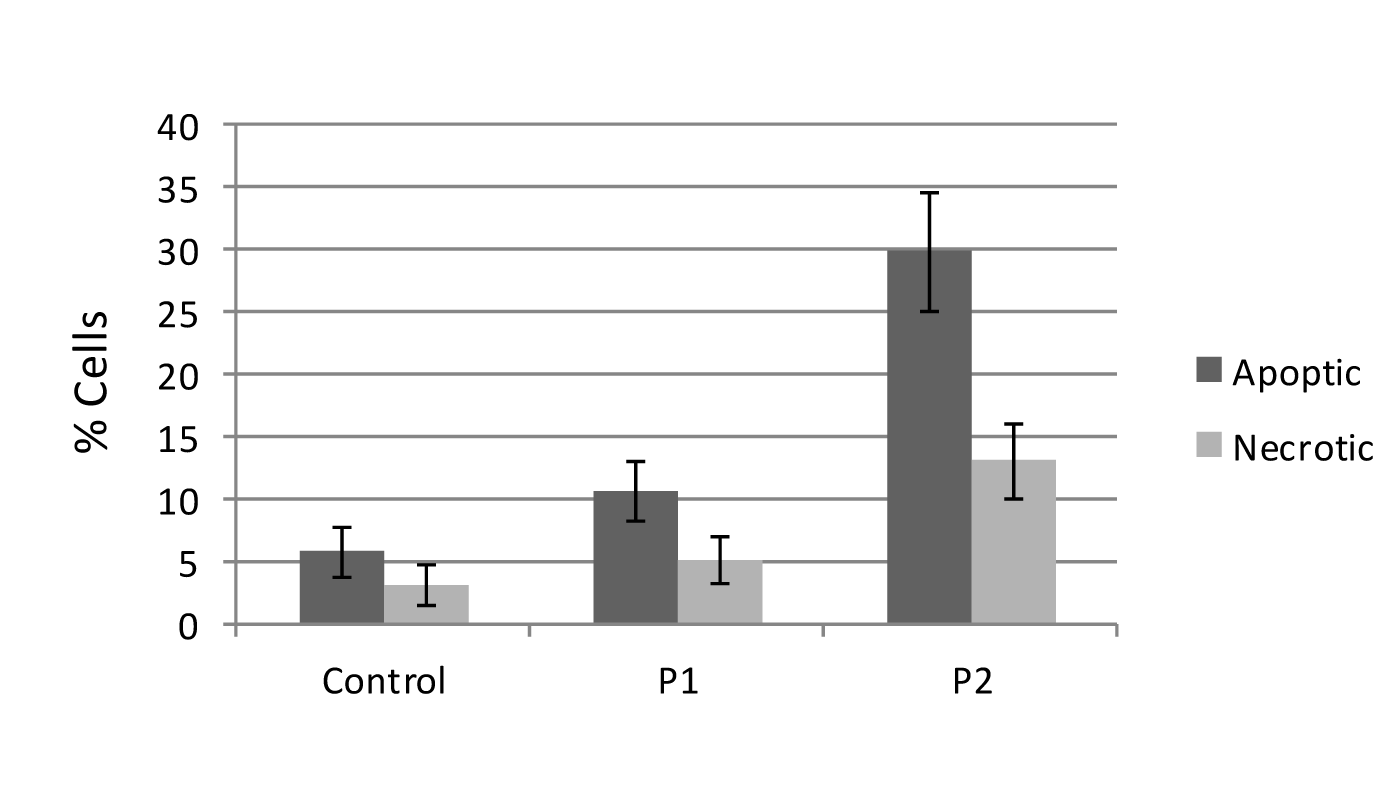

Supplement: Figure S3 — The percentage of apoptotic and necrotic IEC-18 cells co-incubated with P. aeruginosa of the P1 and P2 phenotypes. The counts were normalized to the amount of nuclei stained by DAPI. 4 fields of ∼100 cells imaged from 4 independent dishes/group were included in the quantitative analysis. (TIF) [file pone.0044326.s003.tif]

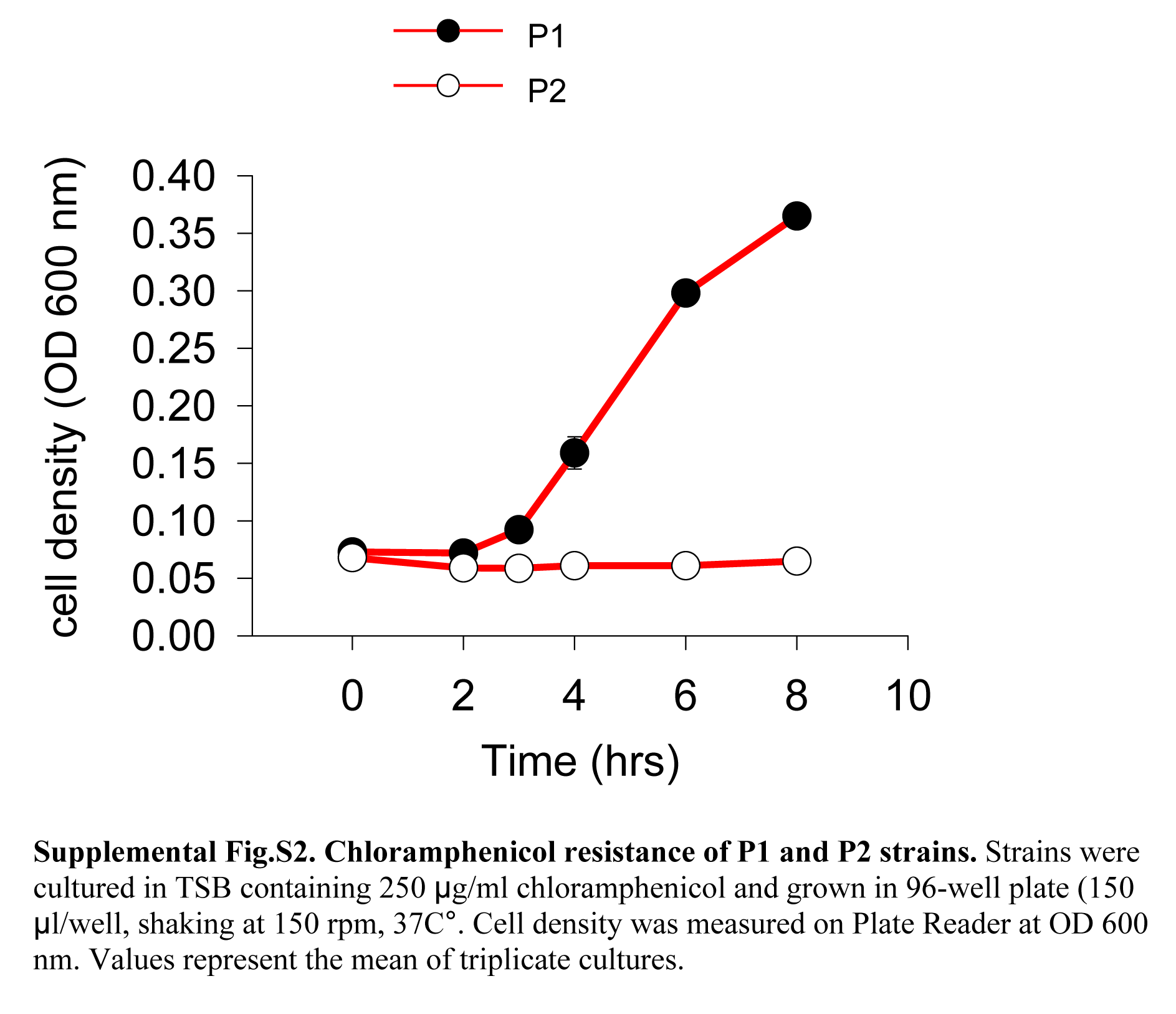

Supplement: Figure S4 — Chloramphenicol resistance of P1 and P2 strains. Strains were cultured in TSB containing 250 µg/ml chloramphenicol and grown in 96-well plate (150 µl/well, shaking at 150 rpm, 37C°. Cell density was measured on Plate Reader at OD 600 nm. Values represent the mean of triplicate cultures. (TIF) [file pone.0044326.s004.tif]
